# Time-Frequency Characteristics of In-Home Radio Channels Influenced by Activities of the Home Occupant

**DOI:** 10.3390/s19163557

**Published:** 2019-08-15

**Authors:** Alireza Borhani, Matthias Pätzold, Kun Yang

**Affiliations:** 1 Faculty of Engineering and Science, University of Agder, P.O. Box 509, 4898 Grimstad, Norway; 2 SuperRadio, Toftes Gate 2, 0556 Oslo, Norway

**Keywords:** non-stationary radio channels, spectrogram analysis, Doppler power spectral density, device-free fall detection, ambient-assisted living

## Abstract

While aging is a serious global concern, in-home healthcare monitoring solutions are limited to context-aware systems and wearable sensors, which may easily be forgotten or ignored for privacy and comfort reasons. An emerging non-wearable fall detection approach is based on processing radio waves reflected off the body, who has no active interaction with the system. This paper reports on an indoor radio channel measurement campaign at 5.9 GHz, which has been conducted to study the impact of fall incidents and some daily life activities on the temporal and spectral properties of the indoor channel under both line-of-sight (LOS) and obstructed-LOS (OLOS) propagation conditions. The time-frequency characteristic of the channel has been thoroughly investigated by spectrogram analysis. Studying the instantaneous Doppler characteristics shows that the Doppler spread ignores small variations of the channel (especially under OLOS conditions), but highlights coarse ones caused by falls. The channel properties studied in this paper can be considered to be new useful metrics for the design of reliable fall detection algorithms. We share all measured data files with the community through Code Ocean. The data can be used for validating a new class of channel models aiming at the design of smart activity recognition systems via a software-based approach.

## 1. Introduction

### 1.1. Background

Global average life expectancy increased by 5 years between 2000 and 2015, and is expected to keep growing globally [1]. According to the World Health Organization [1], around one third of people aged 65+ years fall at least twice a year. Two thirds of those who fall will do so again within six months. The total medical cost of falls is projected to increase from 35 billion dollars in 2013 to over 101 billion dollars in 2030 [2]. Fall incidents come often with serious physical injuries followed by extreme social isolation, if not Kodokushi (long-term undiscovered lonely death). This increases the demand for ambient-assisted living systems, enabling in-home activity monitoring of elderly people so that they can move freely without having to think about wearing sensors.

The global demand for in-home activity recognition solutions has triggered a new approach to health surveillance that uses radio waves reflected off the human body to detect user activity without any need for active user involvement. The new approach is indeed a passive indoor radar solution that transmits radio waves throughout the propagation environment, while fingerprints of the user activities on those waves are collected and processed at the receiver. If an incident occurs, the emitted waves are atypically frequency modulated through the Doppler effect. By using time-frequency signal processing techniques followed by detection algorithms, an emergency message/call can be triggered to inform predefined caregivers. Given the two main categories of fall detection systems [3], namely wearable sensor-based systems and context-aware systems, the radio-frequency (RF)-based system falls into the class of context-aware systems. RF-based systems offer a much cheaper and more functional solutions than its other class members, such as video cameras [4], vision sensors [5], and smart floors [6], which are often expensive and invasive in terms of privacy. Furthermore, RF-based monitoring systems offer a high in-home coverage, thanks to the penetration property of electromagnetic waves. More information on fall detection systems and approaches can be found, e.g., in [7,8,9,10].

There are three main technical steps towards designing an RF-based activity recognition system. Firstly, the channel characteristics that are responsive to the activities of the house occupant must be collected and then pre-processed to denoise the data and to remove irrelevant information. Secondly, sophisticated time-frequency signal processing techniques must be applied to these characteristics to extract the fingerprint information from user activity. Thirdly, robust detection algorithms must be designed to distinguish irregular activities, such as falls, from everyday activities.

This paper addresses the first and the second steps. The focus of the existing literature is often on the third step, while the challenges of the first step are somewhat overlooked. In [11,12,13,14,15,16], the principles of Doppler radars were employed to develop an experimental-based activity recognition system, while in [3], combined range-Doppler features were used to improve the reliability of the detection mechanism. More recently, using a multiple range-Doppler radar for increasing the reliability of the fall detection system was proposed in [17]. The impacts of radar placements on the micro-Doppler and the range intensity have studied in [18]. The WiTrack system uses a frequency modulated carrier wave (FMCW) radar to track three-dimensional (3D) motions of users [19]. This technology was exploited to develop Emerald, which is a preventative product for both detecting falls and monitoring its precursors [20]. In another radar-based experiment [21], two datasets of signatures in different environments have been collected to monitor the daily behavior of individuals at risk of deteriorating physical or cognitive health. In [22], the frequency distribution trajectories associated with falling velocities have been applied to a hidden Markov model to design an experimental-based fall detection system. The signal strength data collected from a radio-frequency identification (RFID) sensor have been employed for activity recognition in [23]. The authors of [24] used an ultra-wide band (UWB) sensor with an operating frequency between 3.1 GHz and 5.6 GHz to detect fall incidents based on the temporal characteristics of the channel. The impact of falls on the temporal properties of the time-variant channel has also been studied in [25].

Halperin et al. have designed a channel state information (CSI) tool that allows for collecting CSI across 30 different subcarriers of the orthogonal frequency-division multiplexing (OFDM) WiFi system [26]. In contrast to the received signal strength indicator (RSSI), the CSI contains both amplitude and phase information across each subcarrier [27,28,29,30]. The WiFall system [31] analyzes the CSI obtained from an off-the-shelf WiFi access point to detect irregular activities in a home setting. The PhaseBeat system uses CSI phase differences to monitor breathing and heartbeat with a commodity WiFi device [32]. The radio tomographic imaging approach has also been used for fall detection purposes. A radio sensor network is often employed to measure the signal attenuation (caused by moving objects) of existing links, while a hidden Markov model is applied to detect irregular variations caused by, e.g., a fall incident [33]. A similar approach has also been applied to RSSI collected from a dense network of WiFi-compliant radio devices installed in a shared workspace [34]. More recently, a real-time system that uses RSSI for sensing human body motions with focus on joint body localization and fall detection has been proposed in [35]. The authors of [36] have proposed an activity recognition system that leverages Doppler characteristics of the channel to detect a fall incident occurred not only after a standing phase, but also after a walking phase. In [37], an intruder detection method was proposed based on the correlation properties of a multiple-input multiple-output (MIMO) channel, while the authors of [38] proposed the antenna arrangement for a 2-by-2 MIMO sensor, evaluating the detection performance based on raytracing simulations. Ikeda et al. proposed an indoor event detection system based on the eigenvector spanning signal subspace obtained from an antenna array [39,40]. Eigenvectors and eigenvalues obtained from this method were then used as features of multiclass support vector machines to classify different states of a human being or an object in an indoor environment, showing that the proposed system can improve localization accuracy compared with RSSI-based activity recognition systems [41,42]. In contrast to a home environment with a single occupant, the authors of [43] analyzed the delay-Doppler properties of an indoor university hall in the presence of many moving people. The authors of [44] provided a comprehensive survey of activity recognition systems based on the aforementioned approaches, while Rucco et al. reviewed the proposed approaches for fall risk assessment, fall prevention, and fall detection for healthy elderly persons [45].

### 1.2. Motivation and Contribution

The RF-based activity recognition approach is still in its early stage of development and requires sufficient fundamental research before the production phase. The majority of the existing literature relies on an experimental-based design approach, which is very time-consuming and costly, as it requires numerous repetitions of the experiment (data collection) in different experimental setups, so that machine learning models can learn sufficiently and can perform reliably. A similar challenge has also been addressed in [46]. However, one can save both time and money with employing a software-based design approach, in which channel models/simulators generate numerous amount of datasets (in almost no time compared to an experiment) that can be used for training and testing detection algorithms under diverse propagation setups and different mobility patterns. To design and parameterize a robust channel model, the physical radio channel and its characteristics (especially from the non-stationarity perspective) need to be thoroughly studied. The software-based design approach has been very rarely, if at all, addressed in the literature. This urged us to conduct a comprehensive indoor radio channel measurement campaign with a reliable channel sounder (rather than an off-the-shelf WiFi card). The collected empirical data can then be used for the development of a new class of channel models aiming at the design of RF-based activity recognition systems. It is noteworthy that the simplified data model (a time-variant complex number) that is often-used in the literature is rather a mathematical formulation of the collected complex CSI, but not a non-stationary channel model that can be used for generating fading behavior. To empower the further development of software-based design approach, we share the collected measurement data with the community through Code Ocean [47]. This allows researchers to better understand the fading behavior of non-stationary indoor channels in the presence of moving objects. In addition, the data can be used for the parameterizations and validation of new channel models (see, e.g., [48,49]) aiming at the design of RF-based activity recognition systems. One can use the data to reconfirm/reproduce the results of this paper.

In particular, this paper reports an in-home channel measurement campaign using a single-input single-output (SISO) radio channel sounder at 5.9 GHz with a bandwidth of 100 MHz. The paper emphasizes the radio characteristics of the channel from the multipath fading and non-stationarity perspectives, showing that a relatively limited radio resource with a single transmit/receive antenna can be sufficient to detect falls even under obstructed line-of-sight (OLOS) conditions. A typical Norwegian house with real-life living conditions is chosen for conducting the measurement campaign. A user performs according to a set of predefined mobility patterns, including the simulation of a fall. The experiment is carried out in both LOS and OLOS propagation conditions. The time-variant channel transfer function (CTF) is recorded, then transformed to the channel impulse response (CIR). To reveal the time-frequency behavior of the channel, a spectrogram analysis is performed on the complex channel gain, estimating the Doppler power spectrum in time. It is demonstrated that the spectrogram not only allows the distinguishing of falls from other activities, such as walking and sitting, but also contains useful information about the mobility pattern (trajectory) of the user. In contrast, it is not straightforward to gain similar information from the time-variant channel envelope. Furthermore, the instantaneous mean Doppler shift and the instantaneous Doppler spread have been estimated from the measured data by means of the spectrogram. It is shown that these two characteristics are clearly impacted by the ongoing activities. Consequently, they can be used as new features for the development of new fall detection algorithms using machine learning [44,50]. We remark that this paper does not aim at designing fall detection algorithms, but studying the physical indoor channel and its characteristics that can be used for fall detection algorithm design purposes. It is shown that the instantaneous Doppler spread filters the details of the ongoing activity, but highlights the coarse variations of the channel, especially if the transmit/receive antennas are under OLOS propagation conditions.

Despite most of the existing literature, this paper justifies the positivity/negativity of the spectrogram results and explain the non-stationary behavior of the empirical data with the theory of the Doppler effect. This is often overlooked in the papers proposing an activity recognition system. The novelty of the paper also arises from the very in-depth analysis of the physical radio channel at a frequency band that has not been studied before (to the best of the authors’ knowledge), providing the researchers in the field with high-resolution empirical data (both in the paper and an online repository) that allow for the development of experimentally driven channel models, while empowering a software-based design approach. Another novel aspect of the paper is the discussion on the non-stationarity level of the instantaneous Doppler spread in different settings, introducing a new detection metric in the context of RF-based activity recognition. This paper does not aim at developing a complete fall detection system, so does not cover all the three developmental steps mentioned above. The focus of the paper remains on the channel characterization from the non-stationarity perspective, while analyzing new time-frequency features. While most of the existing literature has not been supported with theoretical radio channel models, the empirical results presented in this paper have been verified with the channel model proposed in [49].

The remainder of this paper is organized as follows. The radio channel sounder and the experimental setups are introduced in Section 2. Section 3 outlines the theoretical background of the conducted time-frequency analysis. Section 4 illustrates the obtained characteristics and interprets the results, while Section 5 discusses some applications of the results. Finally, Section 6 concludes the paper and outlines future research needs.

## 2. Measurement Methodology

### 2.1. Equipment

A radio channel sounder provided by Super Radio AS (Norwegian University of Science and Technology (NTNU) spin-off) was used to conduct the measurement campaign. The channel sounder employs the time-division multiplexing technique and operates at 5.9 GHz. The signal bandwidth is 100 MHz, allowing for a delay resolution of 10 ns. The transmitter (Tx) is configured to transmit 6187 chirps per second, while each chirp contains 512 samples. The transmit power is 16 dBm, while the antenna gains are 2 dBi. Both the Tx and receiver (Rx) are equipped with single omnidirectional antennas and have been synchronized using the Global Positioning System (GPS) reference clock before recording the channel data. The phase distortion caused by device imperfections is mitigated via a built-in mechanism that is based on the back2back connection of the Tx and Rx.

The time-variant CTF was collected and stored using an Ubuntu 14.04 operating system running on an Intel 2.8 GHz Core i7 processor. Subsequently, the time-variant CIR was obtained by applying the inverse Fourier transform to the time-variant CTF. The complex channel gain was then calculated by integrating over all multipath arrivals (see Section 3.1).

The employed channel sounder was also used in several other indoor and outdoor radio measurement campaigns (see, e.g., [25,51,52]) and has shown a very good performance in capturing the non-stationary properties of channels involving moving scatterers.

The chosen frequency is fortunately close to the WiFi 5-GHz band, which seems to be the future commercial platform of such an in-home healthcare application. This frequency band allows for wave penetration in small concrete apartments, where elderlies probably tend to live. Nevertheless, this experiment was conducted in a wooden Norwegian house, assuring a considerable coverage thanks to the penetration feature of radio waves.

### 2.2. Propagation Environment

**Figure 1:**
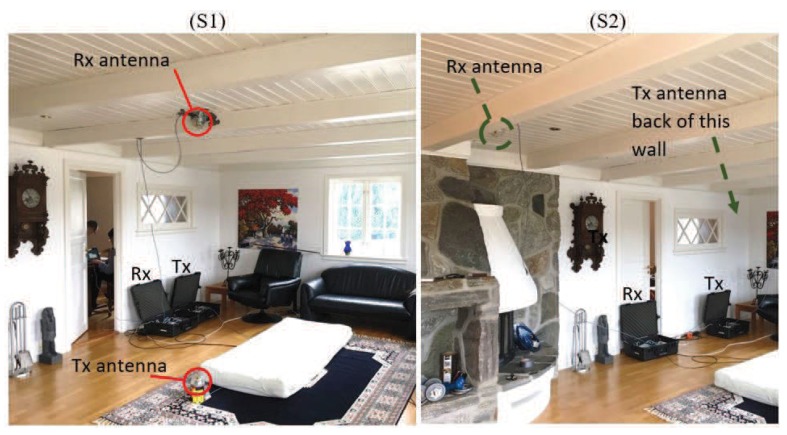
The in-home propagation area, where the measurement campaign was conducted under both LOS (S1, **left**) and OLOS (S2, **right**) conditions.

**Figure 2:**
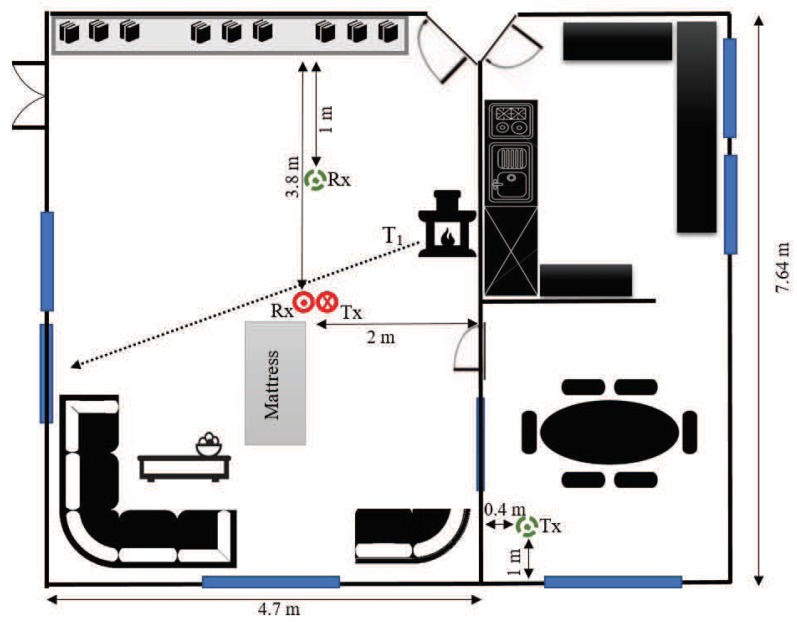
A schematic presentation of the propagation environment, illustrating Trajectory $T_{1}$ of the user, as well as the LOS (red solid-line circles) and OLOS (green dashed-line circles) position of the Tx/Rx antennas.

The campaign was conducted in the living room of a typical two-story Norwegian house located in Grimstad, Norway. The foundation of the building is concrete, while the rest of the structure is mainly wood. The living room is on the first floor with an area of about 36 m${}^{2}$ and a ceiling height of 2.4 m. The room has standard furniture, such as a sofa set, sofa table, fireplace, library, and other furniture. Figure 1 shows parts of the propagation environment, the Tx/Rx system, and the location of the Tx/Rx antennas in two different settings (see Section 2.3). A schematic plot of the measurement setup and the propagation environment is shown in Figure 2. We have presented the position of the Tx/Rx antenna in two different scenarios into this single plot. The figure also demonstrates the approximate trajectory of the user towards an intended window (terminating point) in case that the performer walks safely according to the walking scenario (see Section 2.3).

Five persons were in the house during the measurement. Two of these persons were the system operators and another one was reporting the measurement campaign. These three were asked not to move, yet slow motions were inevitable. A lady on the second floor was engaged in everyday activities. She was asked to reduce her activities, but had more freedom of movement compared to those on the first floor. The fifth person simulated the presence of an independent elderly person moving around the living room according to the predefined action plans (see Section 2.3). Accordingly, the propagation area consisted of many fixed objects, a main moving user (performer), and four slowly moving persons.

### 2.3. Scenarios

Two different settings of Tx and Rx were considered. In the first setting, henceforth called S1, the Tx and Rx were located almost in the middle of the room, but at different heights. The Tx was placed on the floor (0.1 m high), with a LOS to the Rx mounted on the ceiling (2.2 m high). The red solid-line circle ⨀ (⨂) in Figure 2 depicts the position of the Rx (Tx) according to S1.

The second setting, called hereafter S2, was chosen to study the indoor channel under OLOS conditions. Accordingly, Tx and Rx were mounted on the ceiling of the dining room and the living room, respectively. The two areas were separated by a wooden wall that could fairly block the strong LOS component. In Figure 2, the green dashed-line circles display the position of the Tx/Rx in S2. The measurement parameters are briefed in Table 1.

The radio channel measurements have been conducted under different activities performed by a 1.78 m tall, 32-year-old man (user/performer). To prevent injuries, elderly people were not included in the activity plan. The performer was asked to:

(a) *Reference*: Stay out of the living area without any physical activities.
  This scenario gives a benchmark for comparing the properties of the stationary channel with those of the non-stationary channels (associated with the following activities).
(b) *Walking*: Walk slowly along the trajectory $T_{1}$ towards the window (see Figure 2) and stop at the destination point.
  This scenario is designed to analyze the influence of the normal walking of an elderly person on the channel characteristics.
(c) *Falling*: Walk slowly along $T_{1}$ to the mattress, simulate a relatively fast fall (less than a second) on the mattress, then lie motionless on the mattress.
  In this scenario, the impact of rapid movement variations on the channel characteristics is investigated.
(d) *Sitting*: Walk slowly along $T_{1}$ and sit slowly down on a wooden chair (not shown in Figure 1 and Figure 2), where the mattress was previously placed.
  The impact of slow motions of the home occupant on the channel characteristics is studied in this scenario. Moreover, it is intended to see if the impact of the last two action plans on the time-frequency behavior of the channel is distinguishable.

The “start” command (for collecting data) was given by the system operator when the system was ready to collect the data, while the “stop” command was given by the performer when the action was completed. Therefore, both the initial inertia and the terminal inactivity of the performer were captured in the data collection. The CIRs obtained from all scenarios above are provided through Code Ocean [47].

Given the fact that a future radio fall detection system is supposed to support independent living of an elderly, this paper focuses on the impact of a single moving person on the radio characteristics of the in-home channel. However, the study in [53] has stepped further with analyzing the impact of two walking persons on the time-frequency distribution of a similar radio channel.

It is remarkable that the key property, if not disadvantage, of the experimental characterization of radio channels is that the study is site-specific. Repeating the experiment in different settings and propagation conditions (other Tx/Rx settings, walking/falling speeds, more moving persons involved, etc.) is very costly and time-consuming, especially if dedicated channel sounders of high reliability are used. This brings some general applicability concerns for the collected empirical results. This concern can be obviated through years of conducting numerous and comprehensive radio measurement campaigns by independent researchers in the field, such that the experimental findings from such radio environments converge. An alternative approach is to use very low-cost off-the-shelf devices, such as Intel NIC 5300, that allow one to conduct many experiments quickly and inexpensively, but at the cost of a lower data resolution, precision, and accuracy.

## 3. Analysis Methodology

The raw data that the channel sounder measures are the time-variant CTF $H(f^{\prime },t)$ in frequency variable $f^{\prime }$ and the time variable *t*. This section explains the principle of processing the time-variant CTF to derive the channel characteristics.

### 3.1. Complex Channel Gain

The time-variant CIR $h(\tau^{\prime },t)$ can be obtained by taking the inverse Fourier transform of the time-variant CTF $H(f^{\prime },t)$ with respect to $f^{\prime }$. The independent variables $\tau^{\prime }$ and *t* stand for the propagation delay and the time variable, respectively. The corresponding time-variant CIR can be expressed by the following sum

(1) $$\begin{array}{c} h\left(\tau^{\prime },t\right)=\sum_{n=1}^{N}\mu_{n}\left(t\right)\delta \left(\tau^{\prime }-\tau_{n}^{\prime }\left(t\right)\right) \end{array}$$

in which $N=N_{F}+N_{M}$ is the total number of multipath components reflected off $N_{F}$ fixed scatterers and $N_{M}$ moving scatterers. The number of scatterers is indeed the number of delay bins of the discrete channel impulse response after applying the Fourier transform (In the context of radio channel measurement and modelling, this is an assumption on the number of scatterers involved in the propagation mechanism.). The term $\mu_{n}\left(t\right)$ represents the time-variant complex channel gain of the *n*th path, δ(·) denotes the Dirac delta function, and $\tau_{n}^{\prime }\left(t\right)$ describes the time-variant propagation delay associated with the *n*th multipath component.

It follows that the complex channel gain μ(t) of the underlying fixed-to-fixed (F2F) propagation scenario under OLOS conditions can be obtained from (1) by integrating over all arrivals, i.e.,

(2) $$\begin{array}{c} \mu \left(t\right)=\int_{0}^{{}^{\infty }}h\left(\tau^{\prime },t\right)d\tau^{\prime }=\sum_{n=1}^{N}\mu_{n}\left(t\right). \end{array}$$

According to [49], the complex channel gain above can be modelled by a sum $\mu_{F}$ of time-invariant components arriving from $N_{F}$ fixed scatterers and a sum $\mu_{M}\left(t\right)$ of time-variant components arriving from $N_{M}$ moving scatterers. It follows

(3) $$\begin{array}{ccc} \mu (t)\;\;\; & = & \;\;\;\mu_{F}+\mu_{M}\left(t\right)\\ & = & \;\;\;\;\sum_{n_{F}=1}^{N_{F}}c_{n_{F}}e^{j\phi_{n_{F}}}+\sum_{n_{M}=1}^{N_{M}}c_{n_{M}}e^{j\left(2\pi \int_{0}^{t}\;\;\;\;\;f_{n_{M}}\left(t^{\prime }\right)dt^{\prime }+\phi_{n_{M}}\;\right)} \end{array}$$

where $c_{n_{\left(.\right)}}$ denotes the propagation path gains and $\phi_{n_{\left(.\right)}}$ is the phase shift that accounts for the physical interaction of the emitted wave with the $n_{\left(.\right)}$th fixed/moving scatterer (see, e.g., [54] (p. 59)). Please note that under LOS conditions, a single time-invariant component, accounting for the contribution of the shortest propagation path, is added to the sum $\mu_{F}$.

The Doppler shift $f_{n_{M}}\left(t\right)$ caused by the $n_{M}$th moving scatterer is given by

(4) $$\begin{array}{c} f_{n_{M}}\left(t\right)=f_{n_{M},\max}\left(t\right)\left[\cos\left(\Theta_{n_{M}}^{TS}\left(t\right)\right)+\cos\left(\Theta_{n_{M}}^{SR}\left(t\right)\right)\right] \end{array}$$

in which $\Theta_{n_{M}}^{\left(.\right)}\left(t\right)$ is the time-variant spatial angle between the arriving (departing) wave vector at (from) the $n_{M}$th moving scatterer and the velocity of that scatterer. In the equation above, $f_{n_{M},\max}\left(t\right)=\frac{f_{0}}{c_{0}}s_{n_{M}\left(t\right)}$ denotes the time-variant maximum Doppler frequency, where $f_{0}$ denotes the carrier frequency, $c_{0}$ is the speed of light, and $s_{n_{M}}\left(t\right)$ represents the speed of the $n_{M}$th moving scatterer (associated with the performer) along the corresponding trajectory. It is noteworthy that the actual maximum Doppler frequency is $2f_{n_{M},\max}\left(t\right)$, which occurs if $\Theta_{n_{M}}^{TS}\left(t\right)=\Theta_{n_{M}}^{SR}\left(t\right)=0$. Nonetheless, we use the traditional appearance of the maximum Doppler frequency to stay in line with the existing literature.

### 3.2. Spectrogram

A very practical approach to study the time-frequency distribution of non-stationary channels is to perform a spectrogram analysis on the measured complex channel gain. To this aim, μ(t) is first multiplied by a sliding window function $w(t^{\prime }-t)$ to form

(5) $$\begin{array}{c} x\left(t^{\prime },t\right)=\mu \left(t^{\prime }\right)w\left(t^{\prime }-t\right) \end{array}$$

where $t^{\prime }$ represents the running time, *t* denotes the observation time at which the local spectral characteristics of μ(t) is of interest, and w(t) is a positive even window with normalized energy. By applying the Fourier transform to the windowed signal $x(t^{\prime },t)$ with respect to $t^{\prime }$, i.e.,

(6) $$\begin{array}{c} X\left(f,t\right)=\int_{-\infty }^{\infty }x\left(t^{\prime },t\right)e^{-j2\pi \;ft^{\prime }}dt^{\prime }, \end{array}$$

the short-time Fourier transform (STFT) is obtained. The spectrogram $S_{xx}\left(f,t\right)$ is then defined as the squared magnitude of the STFT X(f,t), i.e.,

(7) $$\begin{array}{c} S_{xx}\left(f,t\right)={\left|X(f,t)\right|}^{2} \end{array}$$

which estimates the power spectral density of the Doppler frequencies $f_{n_{M}}\left(t\right)$ in time. Herein, we employ the Gaussian window function w(t) defined as

(8) $$\begin{array}{c} w\left(t\right)=\frac{1}{\sqrt{\sigma_{\omega }}\pi^{1/4}}e^{-\frac{t^{2}}{2\sigma_{\omega }^{2}}} \end{array}$$

where $\sigma_{\omega }$ is called the window spread parameter. This parameter determines the time/frequency resolution of the spectral components of $S_{xx}\left(f,t\right)$. Further details on adjusting the window spread can be found in [55,56], where theoretical insights to the sensitivity of the spectrogram analysis and to the time-frequency trad-off have been provided.

In Section 4, it is shown that the spectrogram $S_{xx}\left(f,t\right)$ reveals the non-stationarity of the Doppler power spectrum and contains useful information about the motion profile of the user.

### 3.3. Instantaneous Doppler Characteristics

The instantaneous mean Doppler frequency $B_{f}^{\left(1\right)}\left(t\right)$ can be obtained from the spectrogram $S_{xx}\left(f,t\right)$ of the complex channel gain μ(t) according to

(9) $$\begin{array}{c} B_{xx}^{\left(1\right)}\left(t\right)=\frac{\int_{-\infty }^{+\infty }fS_{xx}\left(f,t\right)\;df}{\int_{-\infty }^{+\infty }S_{xx}\left(f,t\right)\;df} \end{array}$$

where $S_{xx}\left(f,t\right)$ is given by (7). It is demonstrated in Section 4 that the instantaneous mean Doppler shift $B_{f}^{\left(1\right)}\left(t\right)$ combines the impact of fixed and moving scatterers and gives a tentative figure of the ongoing activity.

The instantaneous Doppler spread $B_{f}^{\left(2\right)}\left(t\right)$ of the non-stationary channel can also be obtained from the spectrogram $S_{xx}\left(f,t\right)$ in (7). It follows

(10) $$\begin{array}{c} B_{xx}^{\left(2\right)}\left(t\right)=\sqrt{\frac{\int_{-\infty }^{+\infty }f^{2}S_{xx}\left(f,t\right)\;df}{\int_{-\infty }^{+\infty }S_{xx}\left(f,t\right)\;df}-{\left(B_{xx}^{\left(1\right)}\left(t\right)\right)}^{2}} \end{array}$$

where $B_{xx}^{\left(1\right)}\left(t\right)$ is the mean Doppler shift in (9). In Section 4, it is shown that the instantaneous Doppler spread $B_{f}^{\left(2\right)}\left(t\right)$ hardly encompasses the details of the performed action, but clearly reveals the major variations in the motion profile of the performer even under both LOS and OLOS conditions.

## 4. Measurement Results

This section is divided into two parts. The first part reports the measurement results associated with S1, where a LOS between the Tx and Rx antennas exists, while the second one explains those of S2, where the communication is established under OLOS propagation conditions. To distinguish between the analytical expressions discussed in Section 3 and the measured channel properties, henceforth, we mark the measured quantities with the star superscript (⋆), highlighting their empirical nature. All the spectrogram plots presented in this section are indeed the top view of the corresponding 3D plots, in which the power of the frequency components is represented by a standard hot color map ranging from black to white (maximum).

### 4.1. LOS Condition (S1)

**Figure 3:**
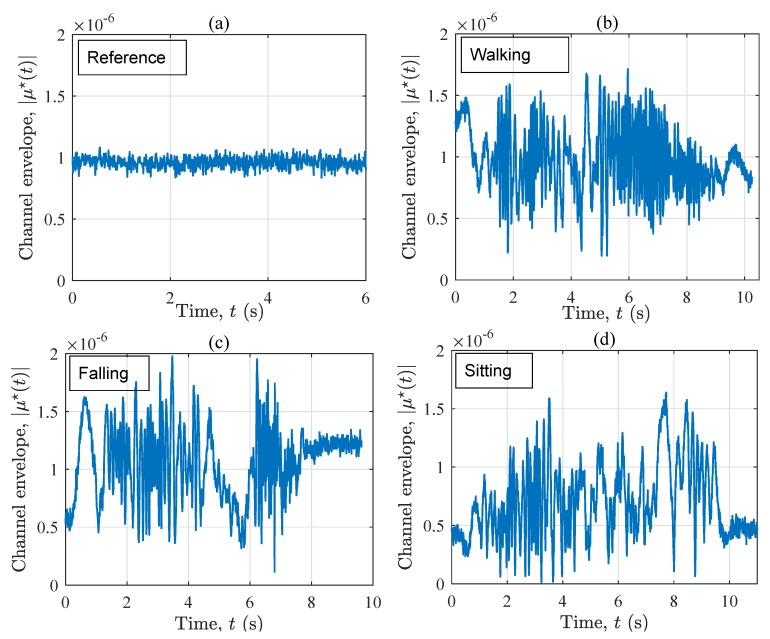
The signal envelope $|\mu^{⋆}\left(t\right)|$ for four scenarios in S1: (**a**) reference, (**b**) walking, (**c**) falling, and (**d**) sitting.

The channel envelope $|\mu^{⋆}\left(t\right)|$ of the measured radio channel corresponding to the four considered activity plans is illustrated in Figure 3. Comparing the four curves, it can be concluded that the variation of the channel in the reference scenario (see Figure 3a) is significantly less than that of the other scenarios (see Figure 3b–d), in which a person moves. Generally speaking, it is hard to recognize a particular activity just by observing the 10-second curves in Figure 3b–d as a whole. Nonetheless, by focusing on particular time frames, let’s say 6<t<7 s, one can see that during a fall (see Figure 3c), the channel envelope fluctuates more than that of the other scenarios. In the next time frame, i.e., 7<t<8 s, these fluctuations start decreasing significantly, as the falling phase is completed, and the user is motionless. Please note that the order of variations in the other scenarios does not change significantly within the same window. For t>8 s, the channel associated with the falling scenario is very stable and similar to that of the reference scenario, but with an additional fixed object, namely the motionless user. From this set of figures, one may conclude that if no prior information about the ongoing activities exists, it is hard to explain the measured channel envelope, as it shows typical small-scale fading behavior. For the same reason, assessing the non-stationarity of the channel in the time domain is not an easy task. This encourages us to study the time-frequency distribution of the channel.

In practice, the measured complex channel gain changes from experiment to experiment even under controlled and similar propagation conditions (activity plans). This is because of microscopic changes in the environment and the status of the hardware, resulting in very diverse phase shifts (modelled by $\phi_{n_{\left(.\right)}}$ in (3)). This makes the design of experiment-based detection algorithm very challenging in the time domain. In contrast, the Doppler spectrum of the channel is not affected by those phase shifts, thus the frequency domain observations allow for more reliable detection algorithm design. In a spectrogram analysis, such phase shifts have no impact on the auto-term (the useful spectral information), but can interfere our time-frequency inspections with cross-terms (artefact of the analysis). It has been shown in [55] that those interfering cross-terms can be removed by applying massive MIMO techniques.

**Figure 4:**
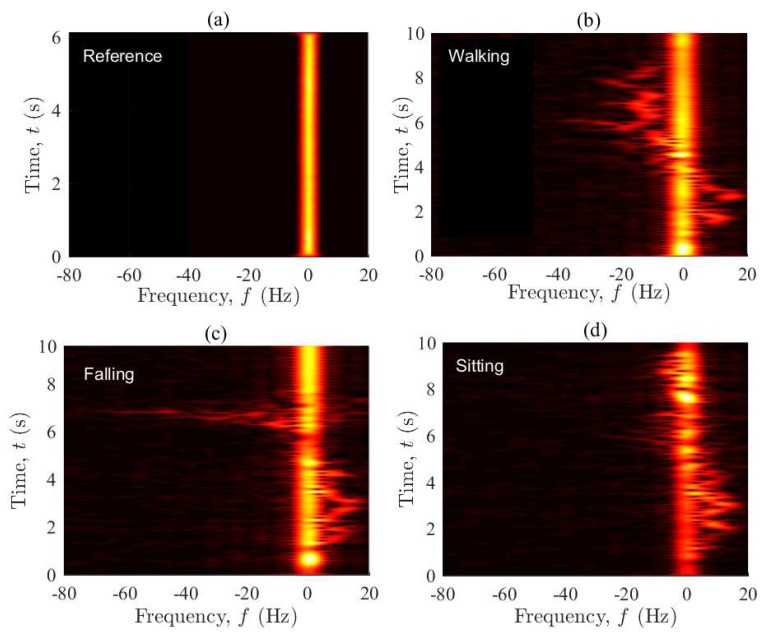
The spectrogram $S_{xx}^{⋆}\left(f,t\right)$ of the complex channel gain $\mu^{⋆}\left(t\right)$ for the four scenarios in S1: (**a**) reference, (**b**) walking, (**c**) falling, and (**d**) sitting.

Figure 4 displays the spectrogram $S_{xx}^{⋆}\left(f,t\right)$ of the measured complex channel gain $\mu^{⋆}\left(t\right)$ associated with the four considered scenarios. The window spread parameter is set to $\sigma_{\omega }=50$ ms (see Section 3.2). The figure shows indeed the distribution of the Doppler frequency components in time. Starting from Figure 4a, the spectrogram of the reference scenario is centered around zero and does not change in time, confirming that the channel is stationary. The zero Doppler frequencies are caused by the fixed objects in the environment, while the near-zero frequencies are mainly due to the limited resolution of the spectrogram analysis (read [55] for the artefact of the spectrogram analysis). Slow movements of the operators’ hands and even their heartbeat can be another reason for the near-zero frequency components. Figure 4b shows both the background time-frequency distribution caused by the fixed objects and the time-variant Doppler shifts due to the walking activity. The plot also reveals the mobility pattern of the performer in the following manner. The person starts accelerating from a zero speed to a maximum walking speed (around 1 m/s), while approaching the Tx/Rx. This results in positive Doppler shifts of about 20 Hz at t=2 s. As the person keeps walking along $T_{1}$ (see Figure 2), the arriving/departing waves become almost perpendicular to the direction of motion, resulting in zero Doppler frequencies at around t=5 s. As the person moves away from the vicinity of the Tx/Rx, the negative Doppler shifts appear. Finally, the deceleration of the performer to a complete stop at the end of $T_{1}$ leads to a zero Doppler shift (caused by the moving person) at about t=9 s. According to Figure 4c, the spectrogram associated with the falling scenario behaves similar to that of the walking scenario within the first six seconds, where the mobility pattern of the performer does not change much. The major difference occurs when the person falls at about t=6.3 s, rising significant Doppler shifts within a very short period of time, i.e., 6.3 s <t<7.1 s. During the falling phase, Doppler shifts of up to −80 Hz occur due to strong changes in the horizontal/vertical speed of the user and the direction of motion. This can also be supported by the video signal processing results reported in [57], showing that for a person walking at 1 m/s in the horizontal plane and 0.1 m/s in the vertical plane (vertical oscillations of the body during a normal walk), both the horizontal and vertical speeds reach near 2.5 m/s if a fall incident happens. The entire process often takes less than a second. Note that the spectral spread of the channel within 6.3 s <t<7.1 s is due to the diverse contributions of different body parts to the Doppler spectrum of the channel. In the sitting scenario (see Figure 4d), the variations of the frequency components within the first five seconds are similar to those of the walking/falling scenario. However, the frequency components are much smaller for the last 5 s, within which the actual sitting takes place at a slow speed. The oscillatory behavior of the spectrogram is caused by the vertical oscillations of the performer during a normal walk. It is remarkable that the first 5 s of the curves in Figure 4b–d are not identical, as the physical channel changes slightly from scenario to scenario. Small changes in the environment (e.g., the placement of a chair or mattress) are unavoidable, while the unknown initial phase of the channel sounder at each record is another reason for minor changes in the results. With reference to the spectrogram plots, it is easy to verify that the channel is non-stationary if the performer moves (even at a low speed) in the propagation environment. This observation was not straightforward to be made in the illustration of the channel envelope.

**Figure 5:**
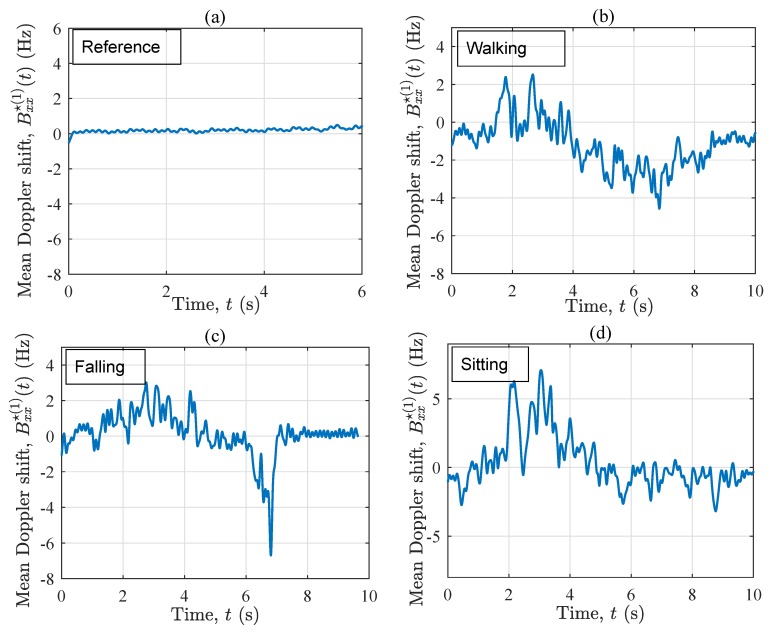
The instantaneous mean Doppler shift $B_{xx}^{⋆(1)}\left(t\right)$ for the four scenarios in S1: (**a**) reference, (**b**) walking, (**c**) falling, and (**d**) sitting.

Figure 5 exhibits the instantaneous mean Doppler shift $B_{xx}^{⋆(1)}\left(t\right)$ pertinent to the four considered scenarios. In the reference scenario, $B_{xx}^{⋆(1)}\left(t\right)$ is rather zero all along the time axis (see Figure 5a. This corresponds to the observations in Figure 4a, where the frequency components are almost evenly centered around the zero frequency all along the time axis. In contrast, when the performer starts walking (accelerating) along $T_{1}$, $B_{xx}^{⋆(1)}\left(t\right)$ starts increasing (through some fluctuations) to about 2 Hz, then decreasing to 0 Hz (see Figure 5b). The latter decrease is because the person approaches the vicinity of the Tx/Rx, where the waves are almost perpendicular to the direction of motion. As the person moves away from the vicinity of the Tx/Rx antenna, the instantaneous mean Doppler shift increases once again, but this time towards negative values. Finally, the deceleration of the performer to a full-stop at the end of $T_{1}$ results in near-zero values of the instantaneous mean Doppler shift $B_{xx}^{⋆(1)}\left(t\right)$. Regarding the fall scenario, a similar trend can be observed in the first five seconds of $B_{xx}^{⋆(1)}\left(t\right)$ in Figure 5c. However, once the fall occurs at about t=6.3 s, $B_{xx}^{⋆(1)}\left(t\right)$ returns sharp negative values down to −7 Hz, which is almost two times larger than the one of the walking scenario at the same time instant. For t>7.1 s, the curves in Figure 5b and c are also very different, as in the first one the performer is still walking, whereas in the other one, the performer lies motionless on the mattress. Figure 5d shows $B_{xx}^{⋆(1)}\left(t\right)$ associated with the sitting scenario. As can be observed from the last five seconds of the curve, even a low-speed sitting action allows for the variations of $B_{xx}^{⋆(1)}\left(t\right)$, distinguishing it from the reference scenario and the “after-the-fall” phase (t>7.1 s) of the falling scenario. The behavior of $B_{xx}^{⋆(1)}\left(t\right)$ in, e.g., Figure 5b agrees with that in Figure 4b. We remind that $B_{xx}^{⋆(1)}\left(t\right)$ is the average of all small and large frequency components, thus its values (e.g., a mean Doppler shift of −7 Hz in a falling scenario) are smaller than the extreme frequency components (e.g., an instantaneous Doppler shift of −70 Hz in a falling scenario caused by one of the fast scatterers) that appear in the spectrogram. Nevertheless, the order of these numbers is still useable for designing a detection algorithm, especially if they are based on machine learning models. Even threshold-based algorithms can be fed with such numbers probably at a cost of a lower detection reliability. Some researchers in the field (see, e.g., [58]) have designed reliable detection algorithms that are based on Doppler shifts in the order of just 0–4 Hz.

**Figure 6:**
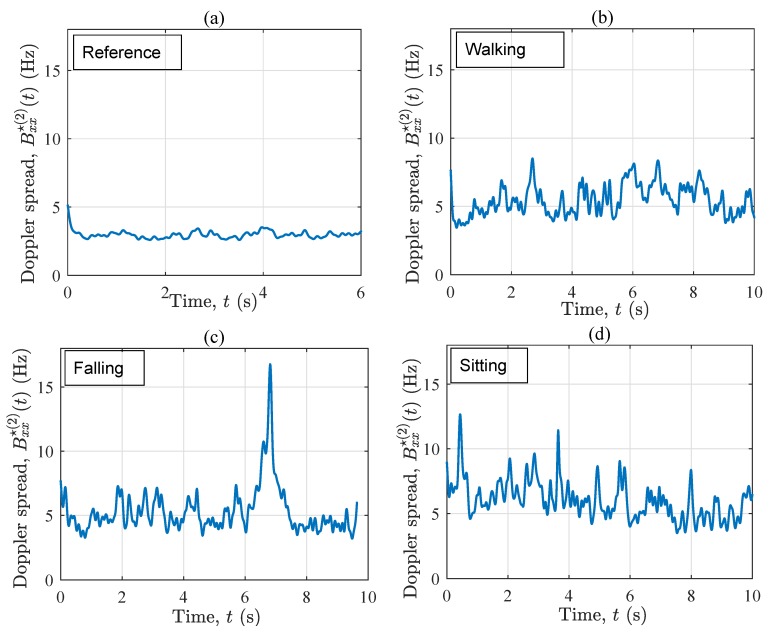
The instantaneous Doppler spread $B_{xx}^{⋆(2)}\left(t\right)$ for the four scenarios in S1: (**a**) reference, (**b**) walking, (**c**) falling, and (**d**) sitting.

Figure 6 demonstrates the instantaneous Doppler spread $B_{xx}^{⋆(2)}\left(t\right)$ associated with the four scenarios. Unlike the instantaneous mean Doppler shift, $B_{xx}^{⋆(2)}\left(t\right)$ contains less information about the mobility pattern of the performer, but this characteristic is clearly fingerprinted by the fall incident as shown in Figure 6c. Comparing Figure 6a with Figure 6b–d, the Doppler spread $B_{xx}^{⋆(2)}\left(t\right)$ varies more in time if the performer is moving in the propagation environment, confirming the non-stationarity of the in-home channel in such cases. Furthermore, $B_{xx}^{⋆(2)}\left(t\right)$ in the reference scenario is slightly smaller (through some fluctuations) than that in the other three scenarios. This is because of the presence of the moving object in the propagation area, as well as the additional cross-terms originating from the moving object. When the fall occurs, $B_{xx}^{⋆(2)}\left(t\right)$ increases to about 18 Hz within less than a second. The rapid growth is due to the sudden appearance of Doppler frequency components that did not exist some milliseconds before the incident. Moreover, the diverse contribution of different body parts within the falling phase is another reason for the growth of the Doppler spread within 6.3 s <t<7.1 s. It can be concluded that the instantaneous Doppler spread is a very good metric for the design of fall detection algorithms, provided that the details of the action are of no interest. In other words, the instantaneous Doppler spread filters out the details of the mobility pattern, such as minor variations in the angle of motion and/or if the user is approaching or leaving the Tx/Rx, but highlights major changes in the speed of the user and/or in its angle of motion.

### 4.2. OLOS Condition (S2)

**Figure 7:**
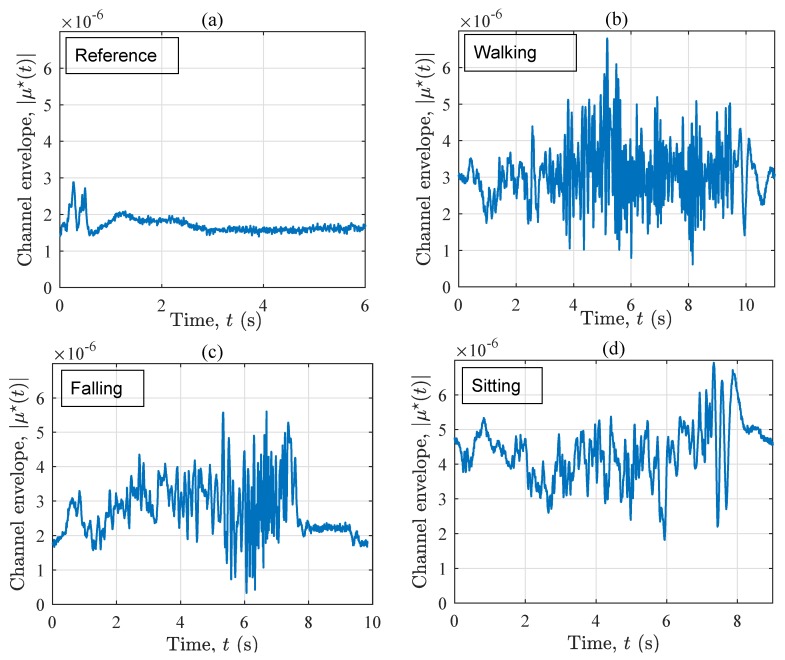
The signal envelope $|\mu^{⋆}\left(t\right)|$ for the four scenarios in S2: (**a**) reference, (**b**) walking, (**c**) falling, and (**d**) sitting.

Figure 7 illustrates the channel envelope $|\mu^{⋆}\left(t\right)|$ associated with the four considered scenarios. In line with our previous observations from S1, the variation of the channel envelope in the reference scenario (see Figure 7a) is much smaller than that of the other scenarios (see Figure 7b–d). According to the last five seconds of $|\mu^{⋆}\left(t\right)|$, the curve in Figure 7c can still be distinguished from that in Figure 7b,d. More especially, the “after-the-fall” phase of the falling scenario, i.e., t>7.1 s, is a large indication for the sudden change of the propagation area from a dynamic one to a fixed one, in which the performer has become motionless. It can be concluded that the channel envelope contains a rough picture of the incident, but not any detailed information about the ongoing activity. To develop a quantified metric for detecting the fall, one idea is to measure the number of level crossings within a moving time window of the channel envelope $|\mu^{⋆}\left(t\right)|$. Nonetheless, we are skeptic of the robustness of this metric because the number of crosses might not be sufficient to draw solid conclusions. Further investigations on this approach is required.

**Figure 8:**
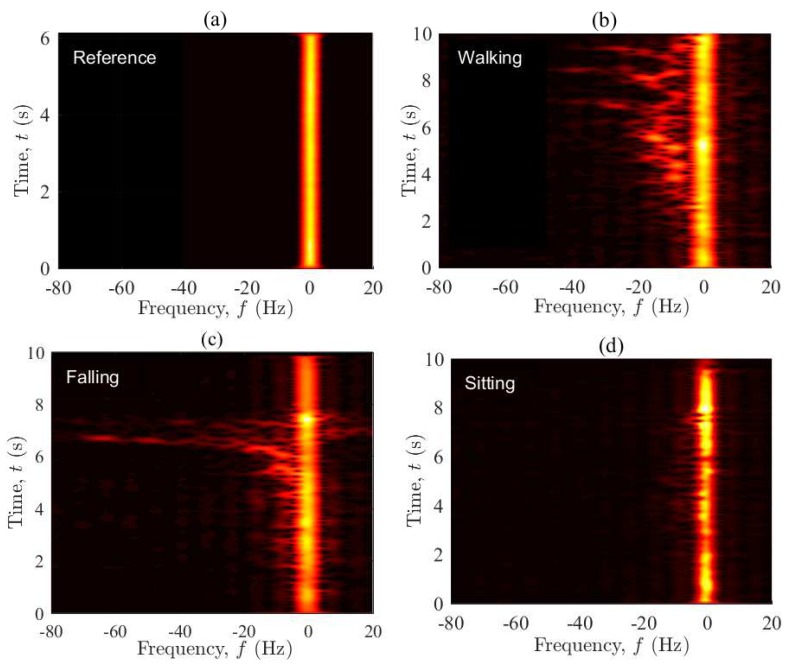
The spectrogram $S_{xx}^{⋆}\left(f,t\right)$ of the complex channel gain $\mu^{⋆}\left(t\right)$ for the four scenarios in S2: (**a**) reference, (**b**) walking, (**c**) falling, and (**d**) sitting.

Figure 8 shows the spectrogram $S_{xx}^{⋆}\left(f,t\right)$ of the process $\mu^{⋆}\left(t\right)$ associated with the four considered scenarios. Each figure estimates the Doppler power spectral density of the corresponding channel in time. The spectrogram of the reference scenario is again time-invariant and zero-centered, confirming that the reference channel is stationary. The reason for the near-zero frequencies is the same as described in the interpretation of Figure 4a. Surprisingly, the mobility pattern of the user can still be tracked even under OLOS conditions when the Tx and Rx were separated in two different rooms. With reference to Figure 8b, the person starts accelerating from zero speed to maximum walking speed while leaving the Tx/Rx (Consider an imaginary line between Tx and Rx in the OLOS setting shown in Figure 2. Walking along $T_{1}$ is then seen as leaving both Tx and Rx.). This increases (through some fluctuations) the Doppler shifts towards negative values, which can be confirmed by the behavior of $S_{xx}^{⋆}\left(f,t\right)$ within the first 8 s of the curve in Figure 8b. While the person is approaching the end of $T_{1}$, he slows down to a zero speed, which finally leads to a zero Doppler shift at about t=9.6 s. The falling scenario can also be tracked without much loss of information due to the OLOS conditions. According to Figure 8c, $S_{xx}^{⋆}\left(f,t\right)$ is similar to that in the walking scenario in the first five seconds and can be explained in the same manner. Once the fall occurs at about t=6.2 s, negative Doppler shifts of down to −80 Hz appear within a second and disappear immediately. For t>7.5 s, the channel then becomes very stable. This latter stationary behavior is because the performer lies motionless on the mattress, while there is no other major moving scatterer in the room. Unlike the results of the LOS setting (S1), the sitting scenario can hardly be traced. With reference to Figure 8d, $S_{xx}^{⋆}\left(f,t\right)$ just reveals that some minor activities have taken place in the propagation area, but no further details.

**Figure 9:**
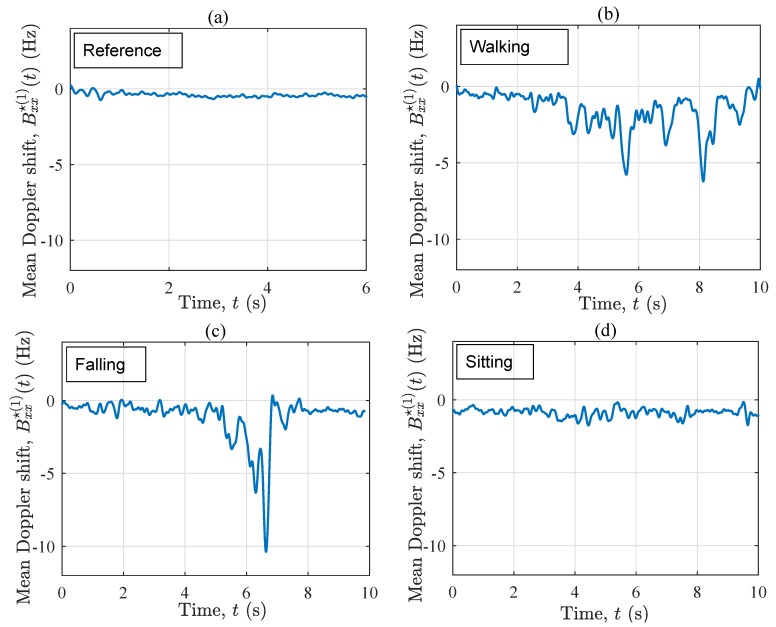
The instantaneous mean Doppler shift $B_{xx}^{⋆(1)}\left(t\right)$ for the four scenarios in S2: (**a**) reference, (**b**) walking, (**c**) falling, and (**d**) sitting.

Figure 9 displays the instantaneous mean Doppler shift $B_{xx}^{⋆(1)}\left(t\right)$ of the four considered scenarios in the OLOS propagation condition. Herein, each curve superimposes (by averaging) the contributions of the fixed and the moving scatterers to the time-frequency distribution of the channel. Accordingly, similar observations can be made as for $S_{xx}^{⋆}\left(f,t\right)$ in Figure 8. The only difference is that the order of the variations is affected by the averaging principles, thus the mean values are smaller than the extreme Doppler shifts seen in the spectrogram $S_{xx}^{⋆}\left(f,t\right)$. For instance, when a fall happens, the spectrogram $S_{xx}^{⋆}\left(f,t\right)$ returns −80 Hz at t=6.2 s, while $B_{xx}^{⋆(1)}\left(t\right)$ returns −11 Hz at the same time instant. Moreover, by comparing Figure 5d and Figure 9d, one can conclude that under OLOS conditions, the instantaneous mean Doppler shift cannot capture the details of low-speed mobility patterns, such as a sitting scenario.

In a nutshell, the instantaneous mean Doppler shift mixes the impact of moving and fixed scatterers on the time-frequency distribution of the channel and delivers useful information about the ongoing activity, but not as detailed as the spectrogram. The mean Doppler shift has clearly indicated the fall incident under both LOS and OLOS settings.

**Figure 10:**
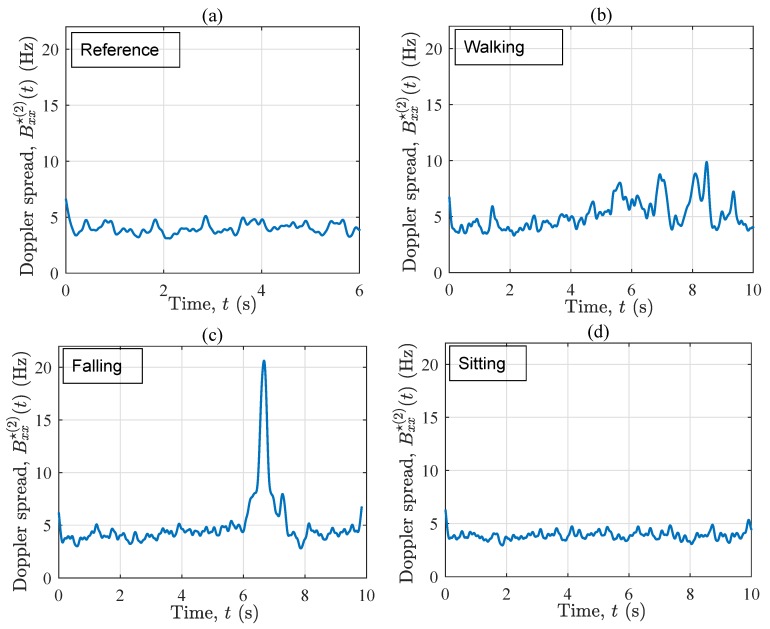
The instantaneous Doppler spread $B_{xx}^{⋆(2)}\left(t\right)$ for the four scenarios in S2: (**a**) reference, (**b**) walking, (**c**) falling, and (**d**) sitting.

Figure 10 exhibits the instantaneous Doppler spread $B_{xx}^{⋆(2)}\left(t\right)$ associated with the four scenarios. Considering the four sub-plots, a key conclusion is that under OLOS condition, $B_{xx}^{⋆(2)}\left(t\right)$ significantly removes the details of the ongoing activity, but captures major variations of the channel. In other words, the instantaneous Doppler spread of the channel is rather stationary if no fall incident occurs. In this context, no particular activity recognition can be made from the first 6 s of $B_{xx}^{⋆(2)}\left(t\right)$ across the four scenarios. Nonetheless, once the fall happens, $B_{xx}^{⋆(2)}\left(t\right)$ increases sharply, so that we can clearly distinguish $B_{xx}^{⋆(2)}\left(t\right)$ in Figure 10c from that in the other three scenarios. This rapid growth happens within a second and is four times larger than the average value of $B_{xx}^{⋆(2)}\left(t\right)$ in all considered scenarios. Accordingly, the instantaneous Doppler spread in an OLOS setting is a very robust detection metric if the purpose is to merely detect fall incidents. Comparing the set of sub-plots in Figure 6 and Figure 10, one might also conclude that the OLOS propagation condition allows for a less fluctuating $B_{xx}^{⋆(2)}\left(t\right)$, which can be a useful property for the design of threshold-based fall detection algorithms.

With reference to Figure 6 and Figure 10, the instantaneous Doppler spread ignores more details of the activity, such as the positivity or negativity of the frequency components, but highlights major changes of the channel caused by the fall incident. In OLOS conditions, the instantaneous Doppler spread is rather stationary in time even when the person walks in the environment. However, if the person falls, the instantaneous Doppler spread increases significantly in both LOS and OLOS conditions. Therefore, the instantaneous Doppler spread can be considered to be a very useful metric for the design of fall detection algorithms, in which the aim is to merely detect fall incidents, but not to perform any detailed activity detection.

## 5. Discussion

A potential use-case of the experimental results presented in this paper is to empower software-based development approach, according to which experimentally verified channel models are designed. An example, the in-home non-stationary channel model proposed in [49] can be used for the development of emerging non-wearable radio fall detection systems under numerous propagation and mobility conditions. The model presented therein consists of a stochastic 3D trajectory model to capture the mobility pattern of the user, a cluster of moving scatterers to account for the body parts of the home user, some fixed scatterers to consider static objects in the room, and a transmitter/receiver to rule the radio communications. The proposed trajectory model was featured with a height reduction mechanism along the planned path, generating the trajectory a forward fall at a random point. The complex channel gain was modelled by a stochastic process, capturing the time-variant Doppler effect caused by the mobility pattern of the home user. That model was validated with the empirical data presented in this paper, showing that the spectrogram and the instantaneous mean Doppler shift of the channel model follows closely those of the physical channel.

It is noteworthy that the spectrogram of the complex channel gain is a very good approximation of the Doppler power spectral density if the spread of the window function is well-adjusted [56]. If so, the interfering cross-terms vanish, thus no averaging mechanism is needed to assure the accuracy of the approximation. This has also been demonstrated in [49], where a close match between the results of the spectrogram analysis and those of the Doppler power spectral density has been illustrated (see and compare Figure 5 and Figure 6, therein). In practice, one does not have multiple copies of the signal to enable the averaging mechanism.

Remarkable is that slow fall incidents and low-speed normal activities, e.g., sitting on a chair, may have very similar impacts on the time-frequency distribution of the channel. It is expected that well-trained machine learning algorithms, which are fed with time-frequency characteristics of the channel, can distinguish those impacts, and thus can reliably recognize the corresponding activity. To this aim, it is recommended that radio channel measurements are video recorded, so the empirical data can be labeled as reference qualifiers. Developing detection algorithms is out of the scope of this paper and requires many more repetitions of different experiments to develop robust algorithms. It is expected that the channel models developed out of this empirical study allow for synthetic data generation as the training/testing input of detection algorithms, reducing the cost (time and money) of experimental data collection campaigns.

The responsiveness of the spectrogram analysis approach to the transmit power (transmission range) has not been studied in this paper, mostly because of the hardware cabling restrictions and room size constraints. Nevertheless, one of our observations was that co-locating Tx and Rx (e.g., both closely mounted on the ceiling) spoils the time-frequency observations. This can be attributed to the fact that in such a tight setting, very dominant radio links mask weaker ones that contain the fingerprint information of the human activity. In this context, the impact of housing environments on the performance of activity recognition systems using Wi-Fi channel state information has been studied in [58].

For comparison reasons, the experiments presented in this paper have been repeated several times. However, the presented results demonstrate a single channel record, while the results of other records follow a very similar trend. It is remarkable that a corresponding real-world system should recognize an activity based on a single real-time noisy dataset. Therefore, it is of interest to analyze the signal under real-world conditions. In addition, averaging over several channel records restricts the theoretical justification of the measured Doppler shifts.

Finally, the authors believe that sophisticated radio channel sounders are superior to the off-the-shelf devices, such as an Intel NIC 5300, if the objective is the thorough characterization of non-stationary radio channels in the presence of moving objects. However, due to their cost, size, and complexity, channel sounders have almost no superiority over the commodity WiFi systems if the immediate goal is to develop an activity recognition system prototype. In fact, sophisticated measurement devices often allow for recording high-resolution high-accuracy channel impulse responses with low phase distortions (caused by device imperfections, such as carrier frequency offset, sampling frequency offset, phase-locked loop offset). They are often supported by robust phase calibration and distortion mitigation techniques that allow one to rely on the measured phase data without any need for postprocessing, which is often required when collecting data using inexpensive commodity WiFi cards. Accordingly, it is recommended that the software-based design approach builds on high-quality data collected preferably from robust radio channel sounders.

## 6. Conclusions

This paper has characterized the time-frequency behavior of an in-home radio channel using experimental data collected from a measurement campaign at 5.9 GHz in the presence of a home occupant. The main goal of the experiment was to study the impact of some daily life activities of an elderly, including a fall incident, on the radio channel characteristics. To this aim, a reference, walking, falling, and sitting scenario were examined under both LOS and OLOS propagation conditions, while a radio channel sounder recorded the CTF. Subsequently, the complex channel gain, spectrogram, instantaneous mean Doppler shift, and the instantaneous Doppler spread of the channel have been derived and studied. It has been shown that under both LOS and OLOS conditions, the channel envelope does not give detailed information about the ongoing activity, but is imprinted by the fall incident, which is shown by higher signal fluctuations within the falling phase. Nonetheless, assessing the non-stationarity of the channel using the time-variant channel envelope is not straightforward. In contrast, it is shown that the spectrogram reveals the detailed time-frequency behavior of the channel, confirming that the F2F channel is non-stationary if the person moves in the environment. The spectrogram contains information about approximate motion directions and speed variations of the user. It has been demonstrated that in case of the fall incident, the spectrogram is fingerprinted with high Doppler frequency components that appear and disappear within a very short period of time. This observation applies to both LOS and OLOS conditions, provided that emitted waves could penetrate through the wooden walls of the home environment. It has been shown that low-speed mobility patterns, such as sitting on a chair, allow for a relatively stationary channel, especially if the Tx and Rx are under OLOS conditions. The instantaneous mean Doppler shift and Doppler spread of the channel have also been studied, showing that they concisely contain the fingerprints of the human activity. The measurement data presented in this paper are shared with the community through Code Ocean. The data can be used not only for reconfirming the results of this paper, but also for the design and validation of a new class of channel models, capturing the impact of moving scatterers on time-frequency characteristics of radio channels. The design of new non-stationary channel models is considered to be a main step towards employing a software-based approach to the design of RF-based activity recognition systems.

The performance of the introduced metrics under OLOS conditions opens a new window to the design and deployment of future fall detection systems, where the rigorous setting of Tx/Rx antennas is obviated. Nevertheless, more channel measurement campaigns, especially in OLOS conditions and at other frequency bands, are required to support our observations. New time-frequency metrics, as new features for machine learning algorithms, need to be designed and tested under different mobility patterns and propagation conditions.

## Figures and Tables

**Table 1:** The measurement parameters.

Parameter	Value/Type
Carrier frequency	5.9 GHz
Chirp bandwidth	100 MHz
Transmitting power at the antenna port	16 dBm
Maximum delay span	25.6 μs
Delay resolution	10 ns
Maximum Doppler shift span	±967 Hz
Number of TX and RX antennas	1
TX and RX antennas beamwidths	omni-direction
Antenna gain	2 dBi
Cable loss in total	6 dB
Temperature	19 ${}^{\circ }$C
